# Supplementary material for: Conditional chemoconnectomics (cCCTomics) as a strategy for efficient and conditional targeting of chemical transmission
Source: eLife. 2024 Apr 30;12:RP91927. doi: 10.7554/eLife.91927 (PMC11060718; doi:10.7554/eLife.91927)
Supplement: Figure 3—source data 3. [file elife-91927-fig3-data3.zip › Figure 3 source data3 part 2.zip!/Figure 3-Source data 3-part2/1053f2-1-CRISPResso_Report_y3hlioj1.zip!/CRISPResso_on_1053f2_2/3b.Insertion_deletion_substitutions_size_hist.pdf]

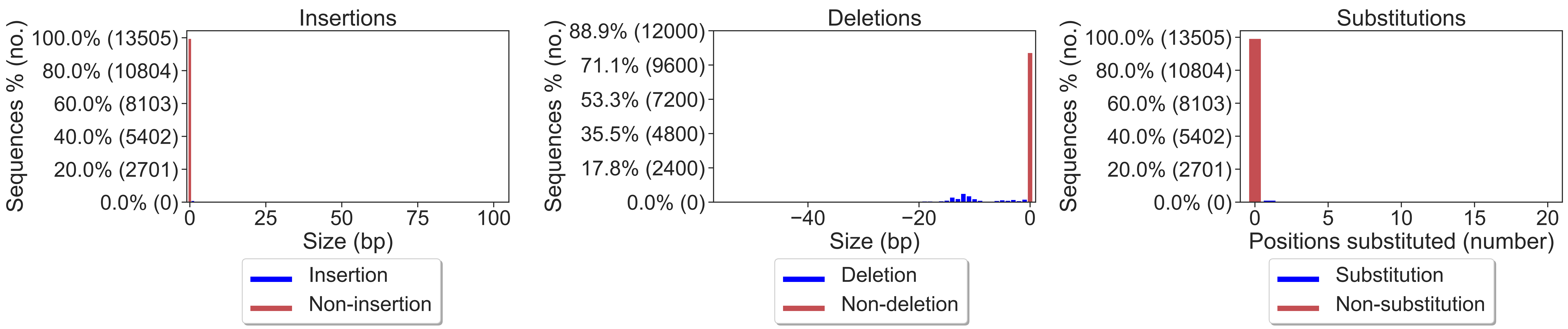

Insertions
Sequences % (no.)
100.0% (13505)
80.0% (10804)
60.0% (8103)
40.0% (5402)
20.0% (2701)
0.0% (0)
0
25
50
75
100
Size (bp)
Insertion
Non-insertion
Deletions
Sequences % (no.)
88.9% (12000)
71.1% (9600)
53.3% (7200)
35.5% (4800)
17.8% (2400)
0.0% (0)
−40
−20
0
Size (bp)
Deletion
Non-deletion
Substitutions
Sequences % (no.)
100.0% (13505)
80.0% (10804)
60.0% (8103)
40.0% (5402)
20.0% (2701)
0.0% (0)
0
5
10
15
20
Positions substituted (number)
Substitution
Non-substitution
